# Unexpected Bone Metastases from Thyroid Cancer

**DOI:** 10.1155/2015/434732

**Published:** 2015-07-05

**Authors:** Sandra Gibiezaite, Savas Ozdemir, Sania Shuja, Barry McCook, Monica Plazarte, Mae Sheikh-Ali

**Affiliations:** ^1^St. Joseph's Regional Medical Center, Department of Medicine, Endocrinology and Metabolism, 703 Main Street, Paterson, NJ 07503, USA; ^2^Department of Radiology, Division of Functional and Molecular Imaging, University of Florida College of Medicine-Jacksonville, Jacksonville, FL 32209, USA; ^3^Department of Pathology and Laboratory Medicine, University of Florida College of Medicine-Jacksonville, Jacksonville, FL 32209, USA; ^4^Department of Medicine, Division of Endocrinology, University of Florida College of Medicine-Jacksonville, Jacksonville, FL 32209, USA; ^5^Endocrinology Fellowship Program, Department of Medicine, Division of Endocrinology, University of Florida College of Medicine-Jacksonville, Jacksonville, FL 32209, USA

## Abstract

*Objective*. To present a complicated case of differentiated thyroid carcinoma (DTC) with metastases to the skull that was evident on I-131 whole body scan (WBS) but negative on other imaging modalities in a low risk patient. *Methods*. We will discuss clinical course, imaging, pathological findings, and treatment of the patient with skull metastasis from DTC. Pertinent literature on imaging and pathology findings as well as radioactive iodine (RAI) treatment impact on quality of life and survival in patients with bone metastases from DTC will be reviewed. *Results*. The patient is a 37-year-old woman with a diagnosis of DTC who had focal areas of increased uptake in the head on WBS with no correlative findings on CT and MRI. Initially, false positive findings were suspected since patient had a low risk for developing metastases. However, the persistent findings on post-RAI treatment WBS, following two courses of treatment, were highly concerning for metastatic bone disease. WBC performed 6 months following the second RAI treatment revealed resolution of the findings. *Conclusions*. False positive findings in WBS are frequent and may be due to contamination, perspiration, or folliculitis of the scalp as well as benign lesions such as meningioma, hematoma, cavernous angioma, and metallic sutures. However, metastatic disease should always be considered even if the patient has low risk of distant metastatic disease and correlative images do not support the diagnosis. RAI therapy appears to improve the survival rates and quality of life of thyroid cancer patients with bone metastases based on retrospective studies.

## 1. Introduction

A small fraction of patients with DTC may present with distant metastases [1, 2]. The figures reported in literature vary from 9% to 15% of patients. The most frequent sites of distant metastases are lungs (50%) and bones (20%). The most frequently involved bones are spine (50%), pelvis (30–50%), ribs (10–30%), femur (20%), skull (10–13%), and humerus (10%) [3].

A complicated case of DTC with metastases to the skull that was evident on WBS but negative on other imaging modalities will be presented. The differential diagnosis of positive bone uptake on WBS but negative on other imaging modalities will be reviewed. In addition, the pathological findings and the relevant literature on prognostic factors and management of metastatic thyroid cancer to bone will be discussed.

## 2. Patient

A 39-year-old female presented to our clinic with complaints of an enlarging lump in the throat. Her family history was significant for thyroid cancer of unknown type in her mother. She had history of right thyroid lobectomy for multiple right lobe nodules at age of 25; one of the nodules was found to be follicular adenoma. At age of 37 she was found to have three nodules in the left lobe. Specimen obtained from one out of three nodules by fine needle aspiration (FNA) at an outside institution was concerning for follicular lesion. Few months later left lobectomy was performed, and surgical pathology specimen review revealed a 6 mm papillary thyroid carcinoma with no evidence of vascular or capsular invasion.

Upon presentation to our institution she had palpable mass in the right side of the neck that corresponded to hypoechoic mass seen in the thyroid ultrasound. It demonstrated increased flow on color Doppler images and was measuring 1.5 × 1.1 × 2.2 cm. An FNA of the mass was performed, but sample obtained was nondiagnostic. The patient elected to proceed with completion thyroidectomy. The pathological diagnosis of this mass was reported as benign thyroid tissue. Given her maternal history of thyroid cancer and also personal history of micropapillary carcinoma, the patient wished to proceed with RAI ablation of remaining thyroid tissue.

**Figure 1:**
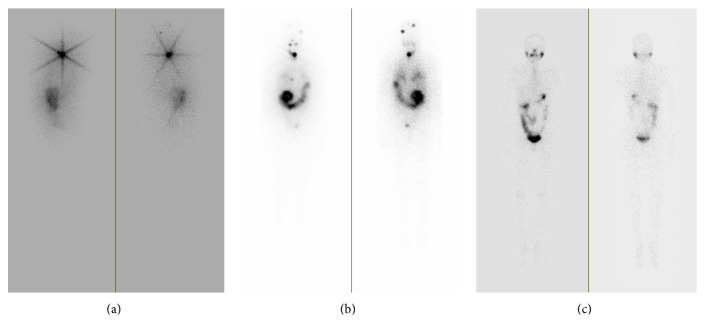
(a) Initial I-131 whole body scan revealing two focal areas of increased uptake in the head. (b) Posttherapy (151 mCi I-131) WBS revealing focal areas of increased uptake in the right anterior chest and left posterior pelvis in addition to previously seen focal areas of increased uptake in the head. (c) Follow-up I-123 whole body scan performed 6 months later demonstrates interval resolution of the previously seen lesions.

The patient underwent a diagnostic WBS five weeks after her last surgery, when thyroid-stimulating hormone (TSH) was 75 mIU/L (0.4–4.5), thyroglobulin (TG) was 65.4 ng/mL (2–35) and the thyroglobulin antibodies (anti-TG) were <20 IU/mL. WBS revealed two focal areas of increased uptake within the left posterior lateral head and mid-portion of the head superiorly (Figure 1(a)). These areas persisted on spot views and repeat images after scrubbing and washing. Head CT with and without IV contrast was performed few days after the WBS and showed no abnormalities in these regions. RAI ablation was performed utilizing 51 mCi of I-131. A posttherapy WBS was performed 7 days later that revealed unchanged uptake in the left parietal and midline occipital region; therefore, an MRI of the brain was recommended. It was performed 13 months later and also showed no abnormalities.

Despite efforts to contact the patient for continuous follow-up, she was not seen in our clinic for the next 15 months; then she returned due to increasing fatigue. She was not compliant with her levothyroxine treatment, which was prescribed after RAI ablation therapy, and her TSH was persistently elevated (ranging between 44 and 48 mIU/L) during the past year. Shortly after visit, a WBS was performed when her TSH was 67, TG was 9.5, and anti-TG were negative. The scan revealed radiotracer activity in the thyroid bed and in the skull, unchanged from the prior scan. Hence, patient underwent a second RAI ablation with 151 mCi. Posttherapy WBS revealed new focal areas of increased uptake in the right anterior chest and in the region of the pelvis (Figure 1(b)). Both lesions were concerning for metastatic disease. A CT scan of chest, abdomen, and pelvis did not reveal any abnormalities that matched the uptake on the WBS. The patient was restarted on thyroid hormone replacement and dose was adjusted to achieve the TSH goal of 0.1–0.3 mIU/L. After six months thyrogen WBS was performed and on day two of thyrogen injection her TSH was >150 mIU/L (0.4–4.5), TG was <0.2 ng/mL (normal range 2–35), and anti-TG were < 20 IU/mL. The WBS showed interval resolution of the previously seen areas of uptake within the skull, chest, and pelvis (Figure 1(c)). 

## 3. Discussion

### 3.1. Pathology Findings

**Figure 2:**
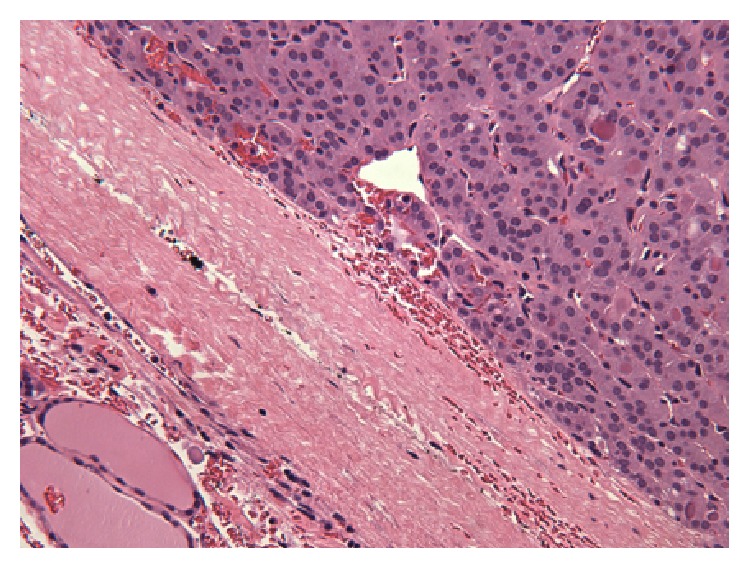
Photomicrograph of thyroid nodule reported as “follicular adenoma” at outside institution in 1996. Encapsulated follicular neoplasm with a thick capsule and microfollicular pattern. No capsular or vascular invasion is seen in this micrograph (hematoxylin and eosin stain; ×200).

As mentioned above, patient had two prior thyroid surgeries at outside institutions. Review of slides from 1996 from outside institution revealed an encapsulated follicular neoplasm with a predominant microfollicular growth pattern (Figure 2). There was no definitive capsular or vascular invasion identified in the tissue submitted for pathological examination. There have been reports of isolated cases in the literature of distant metastases from follicular neoplasms without capsular or vascular invasion, presumably after adequate tumor sampling [4]. However, the sampling of tumor at outside institution in our case was limited with only 4 sections submitted from a 3 cm tumor. Thus, due to limited sampling there is a possibility that a focus of capsular invasion may have been overlooked. There is also the possibility that capsular invasion was missed in the initial histopathological examination rendering an erroneous diagnosis. In a study of minimally invasive follicular thyroid carcinomas from Armed Forces Institute of Pathology, the authors reported that a number of tumors did not show any invasion when sampling constituted only 1 slice per 1 cm of tumor; however, the diagnostic area of invasion was identified when additional sections were submitted to cover greater portions of tumor/capsular interface [5]. Our patient also had a subsequent surgery in 2008 at an outside institution, which revealed a micropapillary carcinoma of 0.6 cm (slides were not available for review). Micropapillary carcinomas are mostly indolent tumors, yet a minority of these tumors do present with distant metastasis. Thus, the two metastatic deposits in the skull could potentially be metastases from either of the two lesions described above, that is, from follicular carcinoma or micropapillary carcinoma. A tissue biopsy of the skull lesions, if clinically indicated, could reveal the histomorphological nature of lesions to document whether they were papillary or follicular. A third subsequent surgery at our institution with removal of residual thyroid revealed benign/hyperplastic thyroid tissue, with no evidence of malignancy.

### 3.2. Imaging Findings

Although brain or skull metastases are not uncommon, the focal areas of increased uptake in the head on initial I-131 WBS were an unexpected finding since the patient had low risk for distant metastatic disease. False positive scans are frequent and may be due to contamination, perspiration, or folliculitis of the scalp [6]. However, persistent finding of focal increased uptake in the same regions on the follow-up study, which was performed more than a year later, would preclude these possibilities. Other causes of false positives include meningioma, hematoma, cavernous angioma, and metallic suture. Again, these abnormalities would likely be identified on cranial CT or MRI. Therefore, the findings were probably due to bone metastasis even though no correlative findings were present on cranial CT or MR likely due to the presence of bone micrometastases. No data is available on the sensitivity and specificity of CT and whole body MRI in screening for bone metastases from differentiated thyroid cancer [7]. Furthermore, resolution of the findings following the administration of 150 mCi I-131 would not be expected in the other etiologies as mentioned above.

### 3.3. Prognostic Factors and Survival

Due to the indolent course of DTC, as well as low rates of bone metastases (BM), the evidence for prognostic factors and outcomes of the management of these patients in current literature is lacking and mainly is based on the retrospective studies. Significant differences in patient selection, staging systems, and clinical management complicate comparison of the available studies.

Most frequently reported positive prognostic factors were absence of nonskeletal involvement [3, 8, 9], treatment with I-131 [3, 9], in particular if cumulative dose of RAI was over 7.4 GBq (200 mCi), BM as revealing symptom of thyroid carcinoma [9], and if BM excision was performed [3]. In a retrospective study of patients with initial BM, 50% of patients younger than 45 years had complete remission following I-131 treatment noting that RAI can be used with curative intent, especially in young patients [10].

Literature reports variable survival rates of patients with BM. French study of 109 patients who received 100–200 mCi RAI after thyroidectomy (87% of patients) or had surgical excision of BM (22%) reported 5-year survival in 41% of patients [8]. Higher 5-year survival rates (53%) were reported in the study of 146 patients from Memorial Sloan Kettering Hospital where 75% of patients were also treated with external beam radiation [3]. Another retrospective analysis of 52 patients reported lower survival rates, which were 36% for 5 years and 10% for 10 years [9]. Interestingly, this study included five cases of micropapillary thyroid cancer, raising concern for aggressive micropapillary thyroid cancer behavior. The more recent study of 106 patients with BM showed the highest 5-year and 10-year survival rates (86.5% and 57.9%, resp.) [11]. This group of patients was treated with multiple RAI therapies utilizing 200 mCi in 4−12-month intervals; also 24.5% of patients had BM excision. The investigators also showed that 39 patients (63.9%) reported the resolution or relief of bone pain after I-131 therapy.

## Abbreviations

DTC: Differentiated thyroid carcinoma
WBS: I-131 whole body scan
RAI: Radioactive iodine
FNA: Fine needle aspiration
TSH: Thyroid-stimulating hormone
TG: Thyroglobulin
anti-TG: Thyroglobulin antibodies
BM: Bone metastases.
